# Puerperal Convulsions

**Published:** 1872-06

**Authors:** Theo. W. Stull

**Affiliations:** Marengo, Ill.


ARTICLE III. *Puerperal Convulsions.* By THEO. W. STULL, M.D., Marengo, Ill.

Puerperal spasm constitutes one of the most fearful complications of the duties and dangers of the lying-in chamber. Their visitations subject the lives of both mother and child to the most imminent peril, and beget excessive fright and consternation amongst friends and attendants. How necessary then that the attending physician should remain calm and collected amid such a scene of confusion and terror. And what are the probabilities of his so conducting himself, except his mind be well stored with a knowledge of the pathology of the disease, and the most approved methods of treatment as well. Bearing in mind that the pathological conditions in the various cases are entirely dissimilar, and that no stereotyped method of treatment is admissible, the routine practice of venesection, used indiscriminately, is as likely to produce harm as good. In the following cases, the treatment was intended to be in accordance with the indications of the pathological condition believed to be present in each case. Whether correct or not, we leave for the reader to judge. During the winter of 1870 and 1871, several cases of the above disease occurred in our vicinity, succeeding each other so rapidly as to almost have the appearance of an epidemic. All the mothers died. All the infants lived, with the exception of one at the seventh month, which was still-born.

Case 1. Was called Dec. 15, 1870, at 4 A. M., to attend Mrs. C. W., who supposed herself taken in labor. She was a strong, healthy, Irish woman, thirty-three years old, and mother of five children. I found her with severe headache, restless, pain in the bowels,—which had given the idea of labor,—scanty and high colored urine, pulse 110, and small. She said she had taken a dose of salts, the day before, which had acted freely. I afterwards found that this was not the case. I informed her she was not going to be confined just now, and thinking the pain in the bowels due to the salts, gave her a powder of morphine and ipecac. to quiet pain and restlessness, and left a solution of nitrate potash and aconite, to be taken hourly for the fever, and returned home. In about an hour I was hastily summoned to return by the terribly frightened husband, who stated that his wife was dying. I hastened to the house again, very correctly interpreting his fright, and found Mrs. W. lying quiet and insensible, and surrounded by several of her excited countrywomen, who had placed lighted candles in the patient's hands, and were most devoutly making innumerable crosses, and reading their Bible to the patient, whose stertorous breathing indicated a somewhat inattentive listener. I told them she was not dying, and directed heat to the extremities, and quiet. An examination disclosed a most rigid os. In a short time she began to moan and showed some signs of returning consciousness, when she was seized with a second fearful convulsion, the interval being about an hour. I now asked that Dr. Hagar be called in counsel, and also sent for chloroform. While we were yet considering what to do, a third convulsion occurred. We now agreed to put the patient fully under the influence of the anæsthetic and maintain it, and also gave two ounces castor oil with two drops croton oil. But the friends strenuously insisted that the chloroform should not be used at all until the priest should arrive, which would be about noon, it now being half past eight. Dr. H. now suggested that we also call Dr. Green to our assistance. He was shortly with us. We now concluded to bleed, when lo! this was not permitted until said priest should also be with us. The doctors now left me until noon when the priest should arrive. The oil was rejected in an hour. Three more spasms occurred during the forenoon, the interval from the last sufficiently long to enable her to answer the questions of the priest quite rationally. Dr. Green now returned, and the priest was scarcely through with the preparation when another seizure occurred. We at once bled to twenty ounces, and had scarcely got the arm bound up when a severe spasm came on. As soon as she could swallow we gave the remainder of the oil. We also directed half-dram doses of potass. bromide, every hour, commencing an hour after the oil was taken. We now left the patient,—I to return as soon as I could visit a very sick patient two and a half miles in the country. At 4 P. M., I was back again, the patient having had two convulsions during the two and a half hours I was absent. But I found that through the influence of some meddlers a homœopath was there. I asked the husband whose patient his wife was, and he said the other fellow's. I had nothing to do but to take my leave. In an hour I was sent for again. I

declined. In a few minutes I was most urgently besought to return to the case by a couple of friends of mine whom the husband had commissioned. I consented, on condition that the "other fellow" be dismissed at once. It was done. I found that the occurrence of three convulsions in a little over an hour, and the last two fifteen minutes apart, was what had settled the other chap. The potash had been discontinued almost from the time I went into the country. The oil had operated freely during the afternoon. I at once gave twenty grains bromide potash, and twenty drops fluid extract gelseminum, with about five drops fluid extract colchicum. In half an hour I repeated the potash, with half the amount of the gelseminum. This was repeated every hour through the night, and at longer intervals during the next day. She had no more convulsions after the first dose, but was soon thrown into the most profuse perspiration, which continued through the night. I gave the medicine myself, as I dare not trust them. The friends importuned me, again and again, to take the child away. I told them if they wanted that done, to get some one else. That was sufficient. Four days later I delivered her of a fine, healthy boy. But she finally succumbed to puerperal fever the third day.

Case 2. Feb. 28, 1871, I was called to meet Dr. Hagar, in consultation, in the case of Mrs. B., who was having convulsions. Mrs. B. was a small, pale, light-complexioned, blue-eyed woman, and mother of three children. She supposed herself seven months advanced in pregnancy. Had suffered, for sometime past, from constipation, severe headache, and scanty urine. Had taken no medicine, being adverse to medication. She was found in the privy, by a neighbor, about 9 A. M., in an unconscious condition, and very cold. How long she had lain there was unknown, but probably a long time, as her fire was out, and quite a pile of snow had drifted upon her dress. Was still unconscious. Had had two or three slight convulsions since being got into the house, and was now quite well warmed up. Upon examination, the os uteri was found pretty firm, though perhaps dilated the size of a goose quill. We were somewhat suspicious of foul play, from exclamations the woman had made to others occasionally. Whilst deliberating what course to pursue, she was seized with a convulsion. We gave two drops croton oil, in an ounce castor oil, as soon as she was able to swallow. I then suggested to the doctor that we give hydrate chloral. Another convulsion. The chloral was sent for, and in twenty minutes the first dose of twenty grains was given. Three minutes after the first dose, another convulsion, which was the last. It occurred at noon. At 2 P. M., the oil moved freely. At 4 P. M., I delivered her of a still-born child, of, I should judge, about seven months, Dr. Hagar being absent. There was no discoverable marks of violence upon the child, which had not probably been dead long. There was but little flowing, the patient lying in a half comatose condition. The pulse began to fail, and I informed the friends that she could not survive long. This surprised them much, as they supposed that delivery ended the danger. Death occurred at 7 P. M.

Case 3. Dec. 7, 1871, was called to attend Mrs. M., who supposed herself taken in labor. She was a small, pale, spare woman, thirty-five years of age, and mother of four children. Symptoms of labor rather slight, when I arrived at 9 P. M. But she was very sure labor would come on before morning. An examination revealed a flabby os, but no dilatation. She had suffered from severe headache and constipation for a long time, but had an inordinate flow of urine. I went to bed and was called at 3 A. M., of the 8th. The patient had an occasional slight pain, and there was a little dilatation. Had had some slight pains during the night. They continued of this character, the woman not suffering much, when suddenly, at 4 o'clock, she was taken with a convulsion. The os was now dilated to the size of a silver dollar, and having the forceps with me fortunately,—for I do not always carry them,—I carefully introduced them and delivered her at once. The uterus contracted well, and there was but little flowing. In half an hour the patient began to recover consciousness a little. A twenty-grain dose of bromide potash was now given. The patient continued to improve, but complained of headache and after-pains somewhat, when, an hour after delivery, she was seized with a second convulsion, severe indeed. I now gave a hypodermic injection, of half a grain of sulphate of morphine. She had no more convulsions, and finally, had a better "getting up" than with either of her other children.

I remark, that in case 1st the cause was evidently centric; the result of uremic poisoning in a robust, plethoric woman. The treatment was, in our opinion, in accordance with the indications of the diagnosis. The result, success as to the convulsions. In case 3d, the cause was undoubtedly eccentric, proceeding from uterine irritation. The remedy, simple and effective. In case 2nd, the cause is not quite so clear, it, perhaps, partaking of both centric and eccentric origin.

